# Effect and Mechanism of Acacetin Against Stress‐Induced Gastric Mucosal Damage: Network Pharmacology, Molecular Docking and Experimental Verification

**DOI:** 10.1002/fsn3.71771

**Published:** 2026-04-12

**Authors:** Xin‐xin Zhang, Lin‐yuan Cao, Fang‐fang Zhang, Shu‐ting Cheng, Qin‐Xin Zheng, Ling Zhu, Zhi‐qiang Zheng, Li‐hong Wan

**Affiliations:** ^1^ Department of Gastroenterology 363 Hospital Chengdu Sichuan China; ^2^ Department of Pharmacology, West China School of Basic Medical Sciences & Forensic Medicine Sichuan University Chengdu Sichuan China; ^3^ NHC Key Laboratory of Chronobiology (Sichuan University), West China School of Basic Medical Sciences & Forensic Medicine West China Second University Hospital, Sichuan University Chengdu Sichuan China; ^4^ Chengdu Experimental Foreign Language School Chengdu Sichuan China

**Keywords:** acacetin, molecular docking, network pharmacology, stress‐induced gastric mucosal damage

## Abstract

Stress‐induced gastric ulcers (SGU) are categorized as a stress‐related mucosal disease (SRMD) that exhibits a high morbidity among ICU patients. However, due to the harmful side effects of proton pump inhibitors (PPIs), there is an urgent need for natural and proactive alternative methods in the treatment of SGU. Acacetin, a natural flavonoid, has demonstrated potential in alleviating gastric ulcers. To elucidate the effects of acacetin in SGU, in this study, gastric ulcers (GU)‐related targets were retrieved from GeneCards, DisGeNET, and OMIM. A protein–protein interaction (PPI) network was constructed, followed by functional enrichment analyses, including Gene Ontology (GO) and Kyoto Encyclopedia of Genes and Genomes (KEGG) pathways. Molecular docking was employed to predict the interactions between acacetin and key target proteins. Furthermore, a cold water‐immersion restraint stress (CWIR) induced mouse model was used to validate the protective effects of acacetin on SGU. Network pharmacology and molecular docking revealed that acacetin exerts mucosal protective effects through multiple ferroptosis‐related targets. In vivo, acacetin significantly mitigated the loss and disorganization of epithelial cells by inhibiting oxidative stress and ferroptosis, indicating its potential to protect against CWIR‐related SGU. Especially, 25 mg/kg/day might be the optimal therapeutic dosage of acacetin as an alternative treatment option for SGU.

## Graphical abstract

This study evaluated Acacetin's therapeutic potential for stress‐induced gastric mucosal damage and investigated its potential mechanism. Based on network pharmacology and molecular docking, Acacetin demonstrated potential mucosal protective effects through multiple ferroptosis‐related targets. In a mouse stress‐induced gastric mucosal damage model, our data confirmed that Acacetin significantly mitigated the loss and disorganization of epithelial cells by inhibiting oxidative stress and ferroptosis.

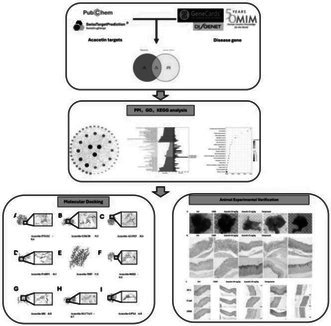

## 1 Introduction

Gastric ulcer is the most common gastrointestinal disease, which is characterized by mucosal damage due to excessive pepsin and gastric acid secretion and a reduction in the protective mucus barrier (Magierowski et al. 2017a). As one of the notable inducers of gastric ulcers, stress is associated with a high mortality rate (Gyires and Feher 2017; Krag et al. 2015). Stress‐induced gastric ulcers (SGU) predominantly occur in critically ill patients or individuals under extreme physiologic stress (He et al. 2022). Currently, proton pump inhibitors (PPIs) are extensively utilized in the prevention of stress ulcers among high‐risk, critically ill patients (Barbateskovic et al. 2019). Unfortunately, PPIs are often used for prolonged periods in an off‐label manner, ultimately resulting in serious, harmful side effects, including an elevated gastric pH, infection, particularly *Clostridium difficile* , and pneumonia and modulation of oral–gut microbiota (Koyyada 2021; Jaynes and Kumar 2018; Zhang et al. 2024). Therefore, there is an urgent need for natural and proactive alternative methods in the treatment of SGU.

Acacetin, also called 5,7‐dihydroxy‐4′‐methoxyflavone, is a natural flavonoid that can be found in over 90 plants, such as Propolis, Agastache rugosa, and Artemisia afra (Singh et al. 2020). Acacetin exhibits a range of pharmacological activities, including antioxidant, antibacterial, and anti‐inflammatory effects (Chen and Gao 2024). Recently, a growing number of studies have underscored the importance of acacetin in inhibiting gastrointestinal inflammation and gastric epithelial apoptosis (Guo et al. 2021; Yao et al. 2024), particularly in cases of alcohol‐ or indomethacin‐induced gastric ulcers (Monforte et al. 2012; Dominguez‐Verano et al. 2025). However, the efficacy of acacetin in treating SGU remains uncertain. Notably, in a mouse model of spinal cord injury—a state of extreme physiological stress—acacetin has been demonstrated to attenuate inflammation and oxidative stress, key drivers of tissue damage, via the Nrf2/HO‐1 pathway (Zhang et al. 2022). Given that the pathogenesis of SGU is similarly driven by inflammatory and oxidative stress responses triggered by stress activation, we reasoned that acacetin might also confer protection against SGU. Furthermore, a growing body of evidence indicates that stress may hinder ulcer healing through psychoneuroimmunological mechanisms (Melinder et al. 2015). Acacetin has exhibited a pronounced reduction in neuronal damage and neuroinflammatory responses (Kim et al. 2012; Ha et al. 2012; Liu et al. 2024). For instance, acacetin was found to suppress lipopolysaccharide (LPS)‐induced production of nitric oxide (NO) and prostaglandin E2 (PGE2) by downregulating the expression of inducible nitric oxide synthase (iNOS) and cyclooxygenase‐2 (COX‐2) in BV‐2 microglial cells (Ha et al. 2012). Notably, the overexpression of COX‐2 has been associated with the pathogenesis of SGU (Magierowski et al. 2017b). Specifically, acacetin has been shown to produce antidepressant‐like effects in murine models by indirectly modulating the activation of 5‐HT1A receptors (Xiao et al. 2019), suggesting its potential role in modulating psychoneuroimmunological pathways. Furthermore, a very recent study demonstrated that acacetin significantly attenuated neuroinflammation and oxidative stress in a murine model of traumatic brain injury (Xie et al. 2022), providing strong in vivo evidence for its ability to protect against stress‐related damage in neural tissues.

Ferroptosis is a form of programmed cell death characterized by mitochondrial damage (Stockwell et al. 2017; Dou et al. 2022), initiated by the accumulation of ferrous iron, which catalyzes lipid peroxidation, ultimately resulting in membrane damage and cell death (Li et al. 2024). Growing evidence highlights the critical role of ferroptosis in the pathogenesis of alcohol‐induced gastric ulcers (GU) (Meng et al. 2024; Zhang et al. 2023). Modern pharmacological studies have confirmed that acacetin can alleviate lipid accumulation and regulate lipid metabolism by suppressing the NRF2/SLC7A11/GPX4 pathway‐mediated ferroptosis (Jiang et al. 2023; Chen et al. 2025). As a medicinal resource with anti‐ferroptotic properties, elucidating these mechanisms of acacetin and refining its application will unleash its full potential in the medical field.

In this study, network pharmacology and molecular docking were employed to predict the target of acacetin in the prevention and treatment of SGU. Furthermore, to validate the gastroprotective efficacy of acacetin, a cold water‐immersion restraint stress (CWIR) model was employed in mice. Subsequently, we analyzed oxidative stress, epithelial integrity, and GPX4 levels in these mice to ascertain the optimal therapeutic dosage of acacetin.

## 2 Materials and Methods

### 2.1 Network Pharmacology Approach

The potential targets of acacetin were identified utilizing the SwissTargetPrediction database (http://www.swisstargetprediction.ch/). Disease targets related to gastric ulcer were retrieved from GeneCards database (https://www.genecards.org/), OMIM (https://www.omim.org/), and DisGENET (https://www.disgenet.org/). The acacetin‐interacting targets and disease‐associated targets were analyzed using Venny 2.1.0 (https://bioinfogp.cnb.csic.es/tools/venny/) to generate diagrams that identify overlapping genes.

GO enrichment analysis, encompassing biological process (BP), cellular component (CC), and molecular function (MF), was conducted on the overlapping targets using the DAVID database (https://davidbioinformatics.nih.gov/). A significance threshold of *p* < 0.05 was applied, and the 20 targets were selected based on their involvement. In addition, KEGG pathway enrichment analysis of the overlapping targets was also conducted under the same significance threshold (*p* < 0.05), with the top 20 pathways being identified. All results were visualized using the online bioinformatics analysis platform (https://www.bioinformatics.com.cn/).

### 2.2 Molecular Docking and PPI

The Retrieval of Interacting Genes/Proteins (STRING) version 12.0 database (https://cn.string‐db.org/) was used to identify potential interacting proteins associated with the overlapped genes of disease‐drug‐ferroptosis, *HOMX1*, *SLC7A11*, and *GPX4*. Molecular docking was performed by CB‐Dock2 (https://cadd.labshare.cn/cb‐dock2/php/blinddock.php) to analyze the binding properties of the ligands for each protein and visualized results with PyMOL 3.1.6.1 (Yang et al. 2022). The 3D structures of proteins were downloaded from the Protein Data Bank (PDB) database (RCSB PDB: Homepage) and exported in PDB format.

### 2.3 Experimental Verification in Animals

#### 2.3.1 Ethics Statement

The animal ethics and experimental protocols were approved by Sichuan University and conducted in accordance with the established guidelines of the China Council on Animal Care (No. K2022005).

#### 2.3.2 Animals and Experimental Groups

Male Kunming mice, weighing between 17–20 g and aged 6 to 8 weeks, were provided from Da‐Shuo Biological Technology Co. Ltd. (Chengdu, China). These specific pathogen‐free (SPF) grade animals were housed under standard laboratory conditions, with five mice per cage, and provided with unrestricted access to food and water. The housing environment was maintained on a 12‐h light/dark cycle (from 8:00 a.m. to 8:00 p.m.), with a controlled temperature of 22°C ± 1°C and relative humidity of 55% ± 10%. All mice were randomly assigned to one of five groups (*n* = 6): control, stress, omeprazole, and two acacetin (Ittabio, Beijing, China, YT62358, 100 mg, HPLC ≥ 98%) treatment groups receiving low (AL) and high (AH) doses. Mice in the omeprazole and acacetin groups were administered 20 mg/kg/day of omeprazole or 25 and 50 mg/kg/day of acacetin, respectively, via oral gavage for five consecutive days. For in vivo administration, acacetin was dissolved in a mixture of 10% DMSO, 40% PEG300, and 50% saline to achieve final doses of 25 and 50 mg/kg, because acacetin has very low water solubility, poor oral absorption, and is unstable under physiological pH and gastrointestinal fluids, resulting in extremely low oral bioavailability (~2%) in rats and rapid plasma clearance after intravenous administration (half‐life ~1.5 h) (Han et al. 2021; Fan et al. 2015; Jang et al. 2025). The stock solution was freshly prepared before use. The stress group received an equivalent volume of saline. On the sixth day, the stress model was induced.

#### 2.3.3 Cold Water‐Immersion Restraint Stress (CWIR) Model

Mice were restrained in polyvinyl chloride tubes and vertically immersed in cold water with a temperature of around 18°C–20°C for 8 h, at the level of the sternum xiphoid. Meanwhile, the mice in the control group were placed in the cages under the same external environment but with no access to water and food.

**FIGURE 1:**
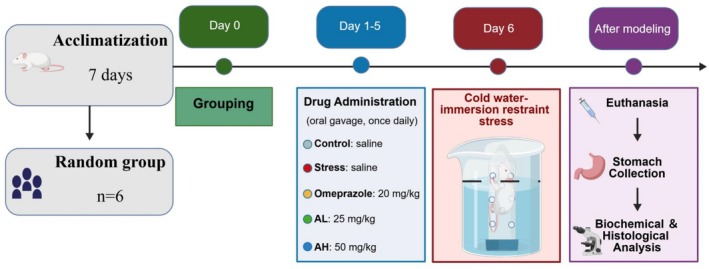
Experimental design and treatment protocol. Mice were acclimatized for 7 days and then randomly assigned to six groups (*n* = 6 per group). Drug administration was performed once daily via oral gavage. Group treatments: Control, saline; Stress, saline; Omeprazole, 20 mg/kg; Acacetin low dose (AL), 25 mg/kg; Acacetin high dose (AH), 50 mg/kg. On day 6, mice were subjected to cold‐water immersion restraint stress. After modeling, mice were euthanized, and stomach tissues were collected for biochemical and histological analyses. This figure was created by BioGDP.com (Jiang et al. 2025).

Following an 8‐h soaking period, all mice were subjected to deep anesthesia via intraperitoneal administration of pentobarbital sodium at a dosage of 50 mg/kg. Once the animals exhibited complete loss of consciousness and absence of response to noxious stimuli, euthanasia was promptly carried out through cervical dislocation. Subsequently, the entire stomach tissue was rapidly removed for subsequent experimental analysis (Ye et al. 2013; Zhang et al. 2016). The outline of the experimental design is shown in Figure 1.

#### 2.3.4 Assessment of Gastric Tissue Injury

The gastric cavity of the mice was opened along the greater curvature and rinsed completely with 5 mL of distilled water. Then, the stomachs were blotted dry with filter paper, spread out, and photographed. The lesion length in the flattened stomach samples was measured using a vernier caliper. The gastric ulcer index (GUI) was calculated as follows: under the dissecting microscope, the amount of bleeding, the size of the ulcers, and their distribution on the gastric mucosa were observed. The longest diameter of each ulcer was measured. Ulcers with the longest diameter ≤ 1 mm were scored as 1 point; those > 1–2 mm were scored as 2 points; those > 2–3 mm were scored as 3 points; those > 3–4 mm were scored as 4 points; and those > 4 mm were scored as 5 points (Liu et al. 2021). Additionally, the ulcer inhibition rate was used to evaluate the degree of gastric mucosal injury, which was calculated as: inhibition ratio (%) = [(GUI_ethanol_ group−GUI_treated_ group)/GUI_ethanol_ group] × 100.

#### 2.3.5 Detection of pH and Pepsin Activity

The pH value of gastric juice was measured by pH test strips.

Approximately 0.1 g of gastric tissue was weighed and homogenized in 1 mL of extraction buffer from the Pepsin Activity Assay Kit (Beijing Solarbio Science & Technology Co. Ltd., China) at 4°C. The homogenate was centrifuged at 10,000 rpm for 10 min, and the supernatant was collected. The protein concentration in the supernatant was determined using the Enhanced BCA Protein Assay Kit (Beyotime Biotechnology, China) at 562 nm. Pepsin activity was measured using the UV spectrophotometry method with the Pepsin Activity Assay Kit. The absorbance of all samples was measured at 275 nm, and the pepsin activity (U g^−1^) was calculated using the formula: Pepsin Activity (U g^−1^) = 0.786 × ΔA ÷ W (Zhou, Wang, et al. 2025).

#### 2.3.6 Hematoxylin and Eosin (HE) Staining and Periodic Acid‐Schiff (PAS) Staining

The gastric tissues were fixed in 4% paraformaldehyde at 4°C for 24 h, embedded in paraffin, and sectioned at a thickness of 4 μm. The sections were stained with HE to observe the pathological morphological changes in the gastric mucosa in a blind study by an experienced pathologist. For each section, 3–5 random fields were selected under a 100× microscope to examine the structure of the gastric mucosa and the infiltration of inflammatory cells in the adjacent tissues. Briefly, it was examined 1‐cm‐long sections for epithelial cell loss (a score of 0–3), presence of inflammatory cells (a score of 0–3), edema in the upper mucosa (a score of 0–4), and haemorrhagic lesion (a score of 0–4) (Yuan et al. 2023).

PAS staining was used to assess the proliferation of goblet cells, quantifying mucus production. The positive area represents the ratio of the area occupied by positive cells to the overall tissue area. The quantification of the positive staining rate in PAS sections was conducted using Image J software (1.54p) (Raish et al. 2021).

#### 2.3.7 Immunohistochemical (IHC)

Gastric tissues were collected from mice and fixed in 10% neutral buffered formalin for 24 h. After dehydration and clearing, the tissues were embedded in paraffin and sectioned into 4‐μm slices, which were mounted on poly‐L‐lysine‐coated slides. The sections were subjected to antigen retrieval by heating in citrate buffer for 15 min in a microwave. Following cooling, endogenous peroxidase activity was quenched with 3% hydrogen peroxide, and the sections were blocked with 5% normal goat serum for 30 min. Primary antibodies (ZO‐1, E‐cadherin, and GPX4) were diluted at 1:100 and incubated overnight at 4°C. Secondary antibodies were applied for 1 h at room temperature. Sections were then stained with 3,3′‐diaminobenzidine (DAB), counterstained with hematoxylin, dehydrated, and mounted. Finally, the sections were observed under a microscope, photographed, and the average optical density was calculated using image analysis software for quantitative analysis (Chang et al. 2025; Zhou, Li, et al. 2025; Li et al. 2025).

#### 2.3.8 Detection of Iron in the Stomachs

The total iron (ferrous and ferric) level in the fresh right portion of gastric tissue homogenates was measured using commercial kits (Beijing Solarbio Science & Technology Co. Ltd., China) at 520 nm with a microplate spectrophotometer reader (SpectraMax190, Molecular Devices, CA).

#### 2.3.9 Determination of Oxidative Stress Level

The levels of superoxide dismutase (SOD) were determined using a Total SOD Assay Kit with WAT‐8 (S0101, Beyotime, China). Malondialdehyde (MDA) levels were tested using a Lipid Peroxidation MDA Assay Kit (S0131, Beyotime, China). SOD levels were measured by the nitroblue tetrazolium (NBT) photoreduction method, while MDA levels were assessed using the thiobarbituric acid (TBA) method (Tang et al. 2024).

#### 2.3.10 Measurements of Glutathione (GSH) Levels

The supernatants of the fresh right portion of gastric tissue homogenates were collected to determine the Glutathione (GSH) levels (Solarbio, China) according to the manufacturer's protocol.

#### 2.3.11 Statistical Analysis

Normality of data distribution was verified using the Shapiro–Wilk test before statistical analysis. All continuous values are expressed as mean ± SD and statistically analyzed by one‐way analysis of variance (ANOVA) with Tukey's HSD correction (GraphPad Prism version 8.0). *p* < 0.05 was considered a statistically significant difference.

## 3 Results

### 3.1 Prediction of Targets and Construction of PPI Networks

**FIGURE 2:**
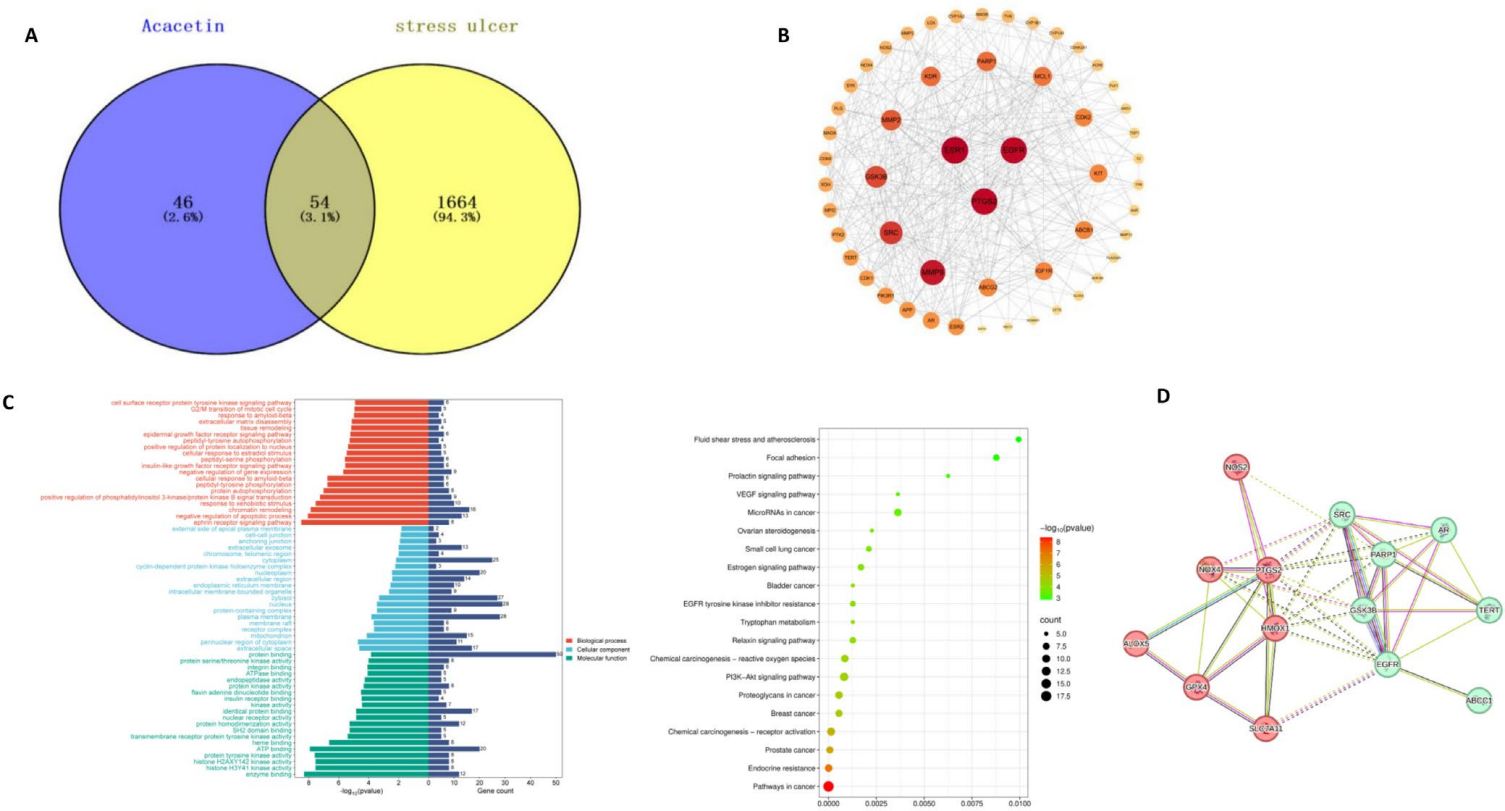
Analysis of target genes in stress gastric ulcers and acacetin. (A) Venn plot showed that within 1718 stress ulcer target genes, 100 acacetin's target genes, and 54 co‐target genes. (B) Protein interaction. Nodes represent relevant targets, and edges indicate protein–protein associations. Number of nodes: 12; number of edges: 57. (C) GO analysis shown in the dot plot, the order is biological process, molecular function, cellular component, and KEGG analysis. (D) PPI analysis of the 11 overlapped genes of Acacetin's therapeutic target in GU and FRGs with GPX4 and SLC7A11 was conducted using STRING.

Initially, 100 target proteins of acacetin were identified from the SwissTargetPrediction database. The GU‐related targets were retrieved from GeneCards, DisGeNET, and OMIM, yielding 1718 disease‐related targets after integration and deduplication. A total of 54 potential targets relevant to acacetin for the treatment of GU were identified by Venn diagram analysis (Figure 2A). To delve deeper into the mechanisms underlying acacetin's therapeutic potential in GU, the 54 shared targets were employed to build a PPI network via STRING (confidence score ≥ 0.4) and visualized by Cytoscape 3.8.0 software. As shown in Figure 2B, the top 3 core genes selected based on degree value screening were *ESR1, PTGS2*, and *EGFR*, which may serve as key targets for acacetin in treating stress‐induced gastric ulcers. GO and KEGG enrichment analysis (Figure 2C) revealed that target proteins were predominantly associated with apoptosis regulation, PI3K signaling transduction, and tissue remodeling. KEGG pathway analysis highlighted the involvement of PI3K/AKT, EGFR, and VEGF pathways.

Next, utilizing data from the ferroptosis‐related genes (FRGs) dataset, a total of 11 overlapping genes related to both acacetin's therapeutic target in GU and FRGs were identified, including *PTGS2, ABCC1, NOX4, GSK3B, ALOX5, EGFR, PARP1, TERT, AR, NOS2*, and *SRC*. As shown in Figure 2D, STRING was employed to establish the PPI network, thereby elucidating the interactions among *GPX4, SLC7A11*, and these overlapping proteins.

### 3.2 Molecular Docking of Acacetin With Key Target Proteins in GU

**FIGURE 3:**
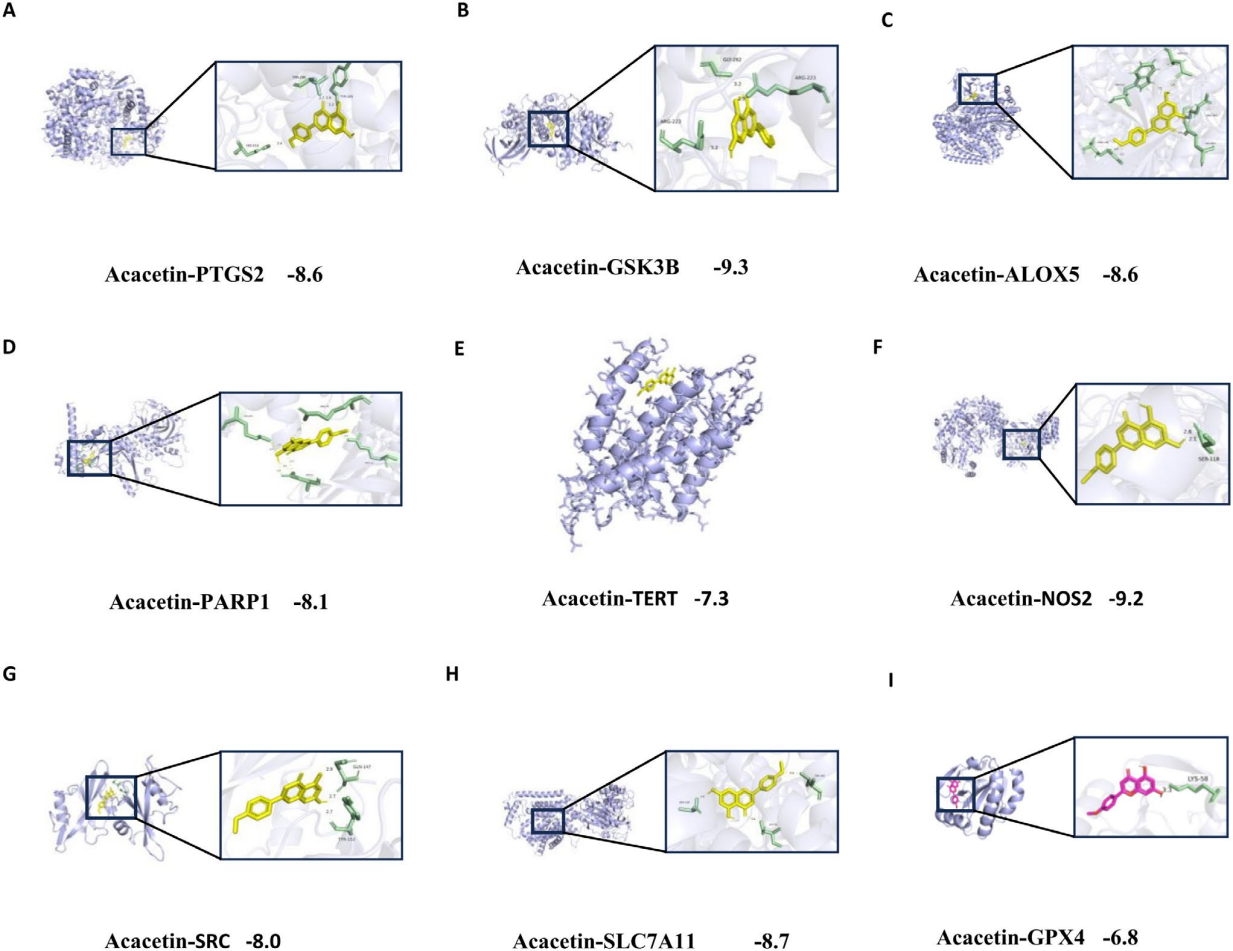
Molecular docking analysis of acacetin with core target proteins associated with GU. (A–I) Molecular docking analysis, the order is PTGS2, GSK3B, ALOX5, PARP1, TERT, NOS2, SRC, SLC7A1, and GPX4.

Based on the previous analysis, *PTGS2, GSK3B, ALOX5, PARP1, TERT, NOS2, SRC, SLC7A1*, and *GPX4* were selected as core targets for molecular docking to verify their interaction activities with acacetin. Interestingly, acacetin had a higher Vina score to all the core targets except TERT (−7.3) and GPX4 (−6.9, Figure 3 and Table 1). The Vina score of *PTGS2, GSK3B, ALOX5, PARP1, SRC, NOS2*, and *SLC7A11* with acacetin was −8.6, −9.3, −8.6, −8.1, −8.0, −9.2, and −8.7, respectively (Table 1).

**TABLE 1:** Molecular docking results of acacetin with core targets.

Gene	PDB	MD score
PTGS2	5f19	−8.6
GSK3B	1h8f	−9.3
ALOX5	3o8y	−8.6
PARP1	4opx	−8.1
TERT	5ugw	−7.3
NOS2	2nsi	−9.2
SRC	1a07	−8.0
SLC7A11	7ccs	−8.7
GPX4	2GS3	−6.8

### 3.3 Validation of the Gastroprotective Effect of Acacetin in a CWIR Mice Model

**FIGURE 4:**
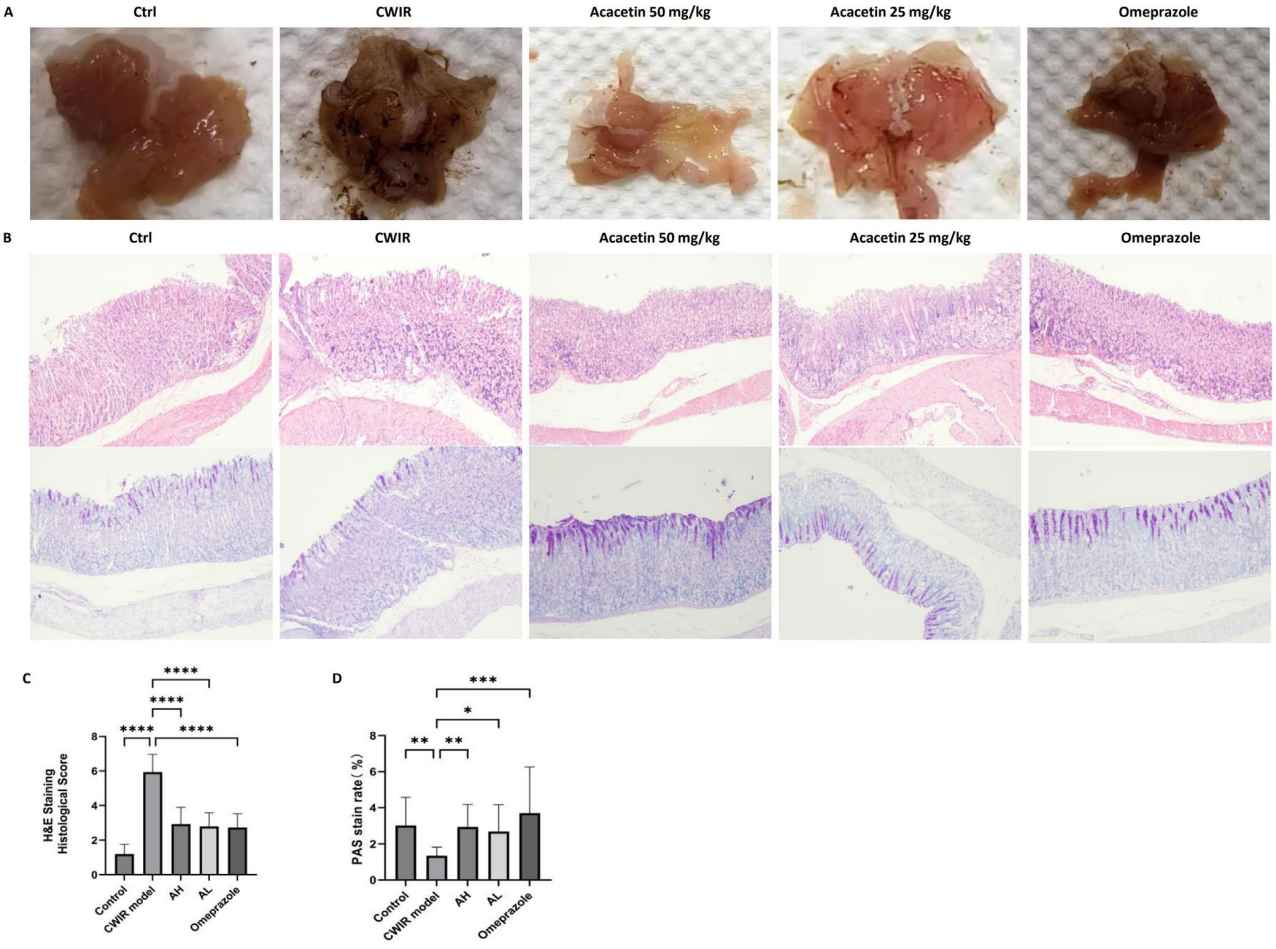
Protective effect of Acacetin on CWIR‐induced gastric ulcer in mice. (A) Gastric tissue images of mice. (B) Represent HE stain in gastric tissues (scale bars: 50 μm) and represent PAS staining in gastric tissues (scale bars: 50 μm). (C, D) Score of HE and PAS. Data are means ± SD and analyzed by Student's *t*‐test or one‐way analysis of variance (ANOVA) with Dunnett post hoc test. All tests were two‐sided (*n* = 5). *p* < 0.05 was considered statistically significant. *p < 0.05, **p < 0.01, ***p < 0.001, ****p < 0.0001, versus the Model group.

The CWIR model of CWIR‐induced gastric mucosal damage was employed to investigate the gastroprotective potential of acacetin. As shown in Figure 4A, the model group showed severe gastric mucosal damage, characterized by linear and spotty bleeding and typical ulceration, as well as the markedly elevated gastric ulcer index (Table 2). Ulcer index differed significantly among groups. One‐way ANOVA revealed a significant difference (*F*(4,25) = 88.72, *p* = 2.1471E−14). Ulcer inhibition rate differed significantly among groups. One‐way ANOVA revealed a significant difference (*F*(4,25) = 434.31, *p* = 1.0546E−22). Notably, the pretreatment with acacetin (50 and 25 mg/kg) and omeprazole elicited a dramatic reduction of ulcer index and a marked increase in the ulcer inhibition rate (Table 2). Gastric pH differed significantly among groups. One‐way ANOVA revealed a significant difference (*F*(4,25) = 6.62, *p* = 0.0009). Moreover, our findings indicated a decrease in pH levels in CWIR mice when compared to the control group, whereas pretreatment with acacetin (50 and 25 mg/kg) and omeprazole significantly reversed this pH reduction (Table 3, *p* < 0.05), suggesting acacetin could effectively inhibit gastric acid secretion and reduce acid‐induced damage to the gastric mucosa. Pepsin activity differed significantly among groups. One‐way ANOVA revealed a significant difference (*F*(4,25) = 3.17, *p* = 0.0309). Additionally, pretreatment with both acacetin and omeprazole resulted in a reduction of pepsin activity (Table 3, *p* < 0.05).

**TABLE 2:** Ulcer index and inhibition rate ($\bar{X}$ ± SD, *n* = 6).

Groups	Dose (mg/kg/day)	Ulcer index	Ulcer inhibition rate (%)
Control	—	0.00 ± 0.00	—
CWIR model	—	60.50 ± 11.36*	0.00 ± 0.00
Acacetin high dose	50	24.67 ± 2.34^#^	58.99 ± 3.89^#^
Acacetin low dose	25	22.83 ± 2.56^#^	62.03 ± 4.26^#^
Omeprazole	20	19.50 ± 4.59^#^	67.58 ± 7.64^#^

*Note:* Compared with the control group.

* *p* < 0.05; compared with the stress model group.

^#^ *p* < 0.05.

**TABLE 3:** Body weight, pH, and pepsin activity ($\bar{X}$ ± SD, *n* = 6).

Groups	Dose (mg/kg/day)	Body weight (g)	pH	Pepsin activity (U/g)
Control	—	18.00 ± 1.10	2.92 ± 0.38	3.70 ± 1.55
CWIR model	—	18.33 ± 1.03	1.67 ± 0.41*	6.56 ± 2.26*
Acacetin high dose	50	18.00 ± 0.63	2.42 ± 0.38^#^	4.04 ± 1.27^#^
Acacetin low dose	25	17.83 ± 1.17	2.67 ± 0.75^#^	4.41 ± 0.86^#^
Omeprazole	20	18.50 ± 0.84	2.75 ± 0.27^#^	4.03 ± 1.66^#^

*Note:* Compared with the control group.

* *p* < 0.05; compared with the stress model group.

^#^ *p* < 0.05.

The histological score differed significantly among groups. One‐way ANOVA revealed a significant difference (*F*(4,70) = 62.92, *p* = 1.873E‐22). As shown in Figure 4B, the CWIR group exhibited extensive gastric wall damage, marked by the loss and disorganization of epithelial cells, coupled with inflammatory cell infiltration within the mucosal layer and interstitial edema. PAS stain rate differed significantly among groups. One‐way ANOVA revealed a significant difference (*F*(4,70) = 4.328, *p* = 0.0035). PAS staining revealed a diminution in purple hue after CWIR, ultimately resulting in the complete depletion of the mucus layer. Pretreatment with acacetin (50 and 25 mg/kg) and omeprazole effectively mitigated the loss and disorganization of epithelial cells, alleviated submucosal edema, and enhanced gastric mucin levels.

### 3.4 Acacetin Mitigated Gastric Mucosa Injury in a CWIR Mouse Model by Inhibiting Oxidative Stress and Ferroptosis

**FIGURE 5:**
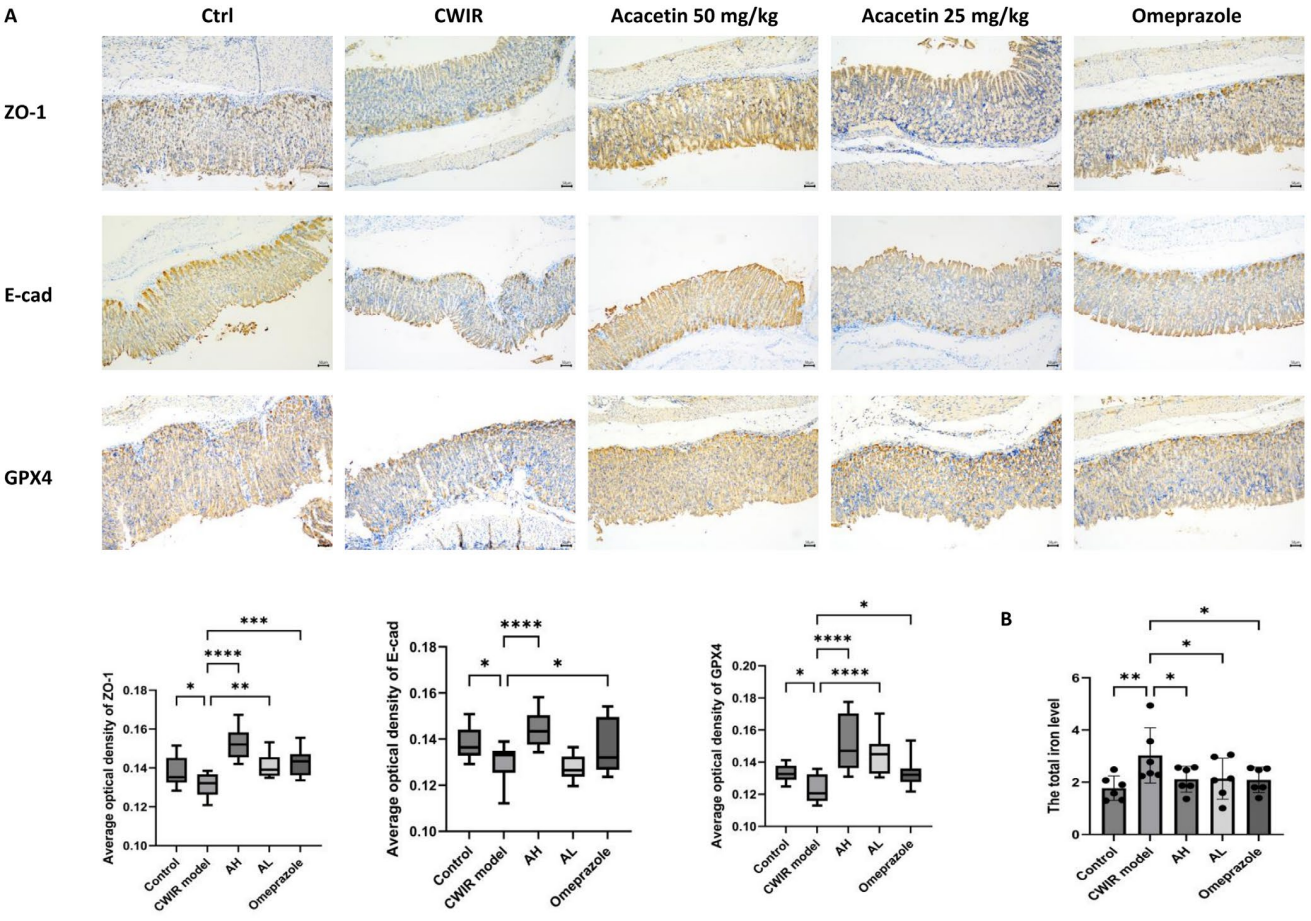
Effect of acacetin on oxidative stress and ferroptosis level in CWIR‐induced gastric ulcer mice. (A) IHC staining for ZO‐1, E‐cad, and GPX4 expression in gastric tissues of CWIR‐induced gastric ulcer mice (upper panel) and the average optical density of ZO‐1, E‐cad, and GPX4 (lower panel). (B) Ferro levels. Data are means ± SD and analyzed by Student's *t*‐test or one‐way analysis of variance (ANOVA) with Dunnett post hoc test. All tests were two‐sided (*n* = 6). *p* < 0.05 was considered statistically significant. *p < 0.05, **p < 0.01, ***p < 0.001, ****p < 0.0001, versus the Model group.

To further evaluate the gastric epithelial barrier function, the expressions of tight junction proteins (ZO‐1 and E‐cad) in the gastric tissues of mice were quantified by IHC. As shown in Figure 5A, ZO‐1 and E‐cad were diminished in the CWIR group but rescued by acacetin (50 and 25 mg/kg) and omeprazole treatment. To confirm whether ferroptosis is the critical mechanism of gastric epithelial barrier damage, the levels of GPX4 and total iron were evaluated. GPX4 staining intensity differed significantly among groups. One‐way ANOVA revealed a significant difference (*F*(4,60) = 13.88, *p* = 4.5137E−8). As expected, GPX4 staining intensity was notably lower in the CWIR groups compared to the control group, which was significantly reversed by acacetin (50 and 25 mg/kg) and omeprazole treatment (Figure 5A). Although the overall one‐way ANOVA did not reach statistical significance (*F*(4,25) = 2.70, *p* = 0.0536), post hoc comparisons revealed that the iron levels in the CWIR groups were significantly higher than those in the control group, while acacetin (50 and 25 mg/kg) and omeprazole significantly reduced them (Figure 5B).

Oxidative stress is widely recognized as a trigger of ferroptosis (Yu et al. 2021). SOD differed significantly among groups. One‐way ANOVA revealed a significant difference (*F*(4,25) = 6.65, *p* = 0.0009). GSH differed significantly among groups. One‐way ANOVA revealed a significant difference (*F*(4,25) = 3.52, *p* = 0.0206). MDA differed significantly among groups. One‐way ANOVA revealed a significant difference (*F*(4,25) = 3.77, *p* = 0.0156). Regarding oxidative stress markers, compared with the control mice, the levels of SOD and GSH (antioxidant enzymes) in the gastric tissue of the CWIR mice were significantly reduced, and the level of MDA (a lipid peroxidation product) was significantly increased (*p* < 0.05, Table 4), indicating increased oxidative stress in the gastric tissues. In contrast, acacetin (50 and 25 mg/kg) and omeprazole application significantly increase the levels of SOD and GSH, and reduce the level of MDA in gastric tissue (*p* < 0.05, Table 4).

**TABLE 4:** SOD and MDA ($\bar{X}$ ± SD, *n* = 6).

Groups	Dose (mg/kg/day)	MDA (nmol/mg prot)	SOD (U/mg prot)	GSH (μg/mg prot)
Control	—	1.97 ± 0.39	24.55 ± 3.50	39.76 ± 2.43
CWIR model	—	3.25 ± 0.84*	10.67 ± 2.36*	26.35 ± 1.15*
Acacetin high dose	50	2.40 ± 0.79^#^	17.27 ± 4.46^#^	39.21 ± 7.71^#^
Acacetin low dose	25	2.41 ± 0.47^#^	16.70 ± 7.44^#^	37.46 ± 10.26^#^
Omeprazole	20	2.11 ± 0.51^#^	19.01 ± 4.33^#^	35.91 ± 9.07^#^

*Note:* Compared with the control group.

* *p* < 0.05; compared with the stress model group.

^#^ *p* < 0.05.

## 4 Discussion

SGU is categorized as a stress‐related mucosal disease (SRMD) that exhibits a high morbidity among ICU patients (Saxena and Singh 2017). The CWIR model is widely used to induce acute stress models in rats (Chen, Xie, et al. 2022) and in mice (Lopes et al. 2023). In our preliminary study, we found that immersing mice in water at 18°C to 20°C for 8 h could establish a stable gastric mucosal injury model, mainly characterized by linear and punctate hemorrhages.

As a natural flavonoid (Singh et al. 2020), acacetin exhibits a wide array of pharmacological activities while possessing exceptionally low toxicity levels, not exceeding 50 mg/kg (Chen and Gao 2024). In a previous study, acacetin was found to inhibit gastrointestinal inflammation and gastric epithelial apoptosis (Guo et al. 2021; Yao et al. 2024) and protect against alcohol‐ or indomethacin‐induced gastric ulcers (Monforte et al. 2012; Dominguez‐Verano et al. 2025). However, its role in SGU has not been clearly studied yet. To explore the intricate interplay between acacetin, its targets, and associated diseases, we initially utilized network pharmacology to dissect the functional role of acacetin in gastric ulcers, drawing upon extensive bioinformatics data. In our study, we collected acacetin targets from the SwissTargetPrediction databases. A total of 100 acacetin‐related targets were identified. A total of 1718 targets for GU were collected from the GeneCards, DisGeNET, and OMIM databases. There were 54 targets in the overlapping region between acacetin and GU. The top 3 core target genes were ESR1, PTGS2, and EGFR, indicating the potential role of acacetin in anti‐inflammatory processes and epithelial repair.

The mucosa is the body's initial barrier against external pathogens and allergens. This gastric mucosal barrier consists of a densely packed layer of epithelial cells, a specialized mucus coating, and bicarbonate ions. The disruption of this barrier likely serves as the primary pathological factor in SRMD, potentially enhancing permeability and initiating immune activation (Yu et al. 2021). Typically, the maintenance of these physical and chemical barriers is aided by specialized epithelial cells, notably gastric epithelial cells (GECs), that establish tight junctions and produce a multitude of barrier proteins, including claudins, occludin, and zonula occludens (ZO)‐1 (Wizenty et al. 2020). ZO‐1 plays a crucial role in establishing epithelial polarity and facilitating the organized formation of apical junctional complexes (Otani et al. 2019). Consistent with the previous findings (Liang et al. 2025), a reduction in ZO‐1 levels was observed in the CWIR mice mucosa, which were partially restored after acacetin treatment in the present study. Moreover, E‐cadherin (E‐cad), the primary component of adherens junctions (AJs), is crucial for maintaining cell–cell contacts within the epithelial cells of the stomach (Kahtan Al‐Bayaty et al. 2019). In this study, we observed a similar trend in the change of E‐cad and ZO‐1 levels between CWIR mice and mice treated with acacetin. Collectively, these results suggest that acacetin plays a pivotal and protective role in safeguarding gastric mucosal defense mechanisms against SGU, potentially by strengthening the integrity of the epithelial barrier.

Currently, ferroptosis of gastric mucosal epithelial cells has been verified to directly lead to disruption of tight junction structures, subsequently triggering alcohol‐induced ulcer formation (Meng et al. 2024; Zhang et al. 2023; Chen, Zhang, et al. 2022). By integrating the FRGs dataset with networking pharmacology, we successfully identified a total of 11 overlapping genes that are pertinent to both acacetin's therapeutic target in GU and FRGs. Importantly, the results of molecular docking revealed that PTGS2, GSK3B, ALOX, PARP1, TERT, NOS2, and SLC7A1 emerged as potential direct targets of acacetin. Furthermore, we observed that elevated iron levels and diminished GPX4 expression were present in CWIR mice, whereas acacetin proved efficacious in mitigating iron overload and upregulating GPX4 expression in the gastric tissue of CWIR mice. This is consistent with previous reports that acacetin can alleviate lipid accumulation and regulate lipid metabolism by suppressing the NRF2/SLC7A11/GPX4 pathway‐mediated ferroptosis (Liu et al. 2021; Yu et al. 2021). As a trigger of ferroptosis (Yu et al. 2021), gastric tissue oxidative stress levels, including the lipid peroxidation byproduct MDA, and antioxidant enzymes SOD and GSH, were evaluated. As expected, acacetin application significantly increases the levels of SOD and GSH and reduces the level of MDA in gastric tissue. All these results confirmed the inhibitory effect of acacetin on oxidative stress‐triggered ferroptosis in SGU mice.

Notably, this study revealed no significant differences between acacetin and the positive control drug omeprazole, hinting at acacetin's potential as an alternative treatment option for SGU. Moreover, there were no significant differences between high and low doses of acacetin, indicating that 25 mg/kg/day might be the optimal therapeutic dosage of acacetin.

## 5 Conclusions

**FIGURE 6:**
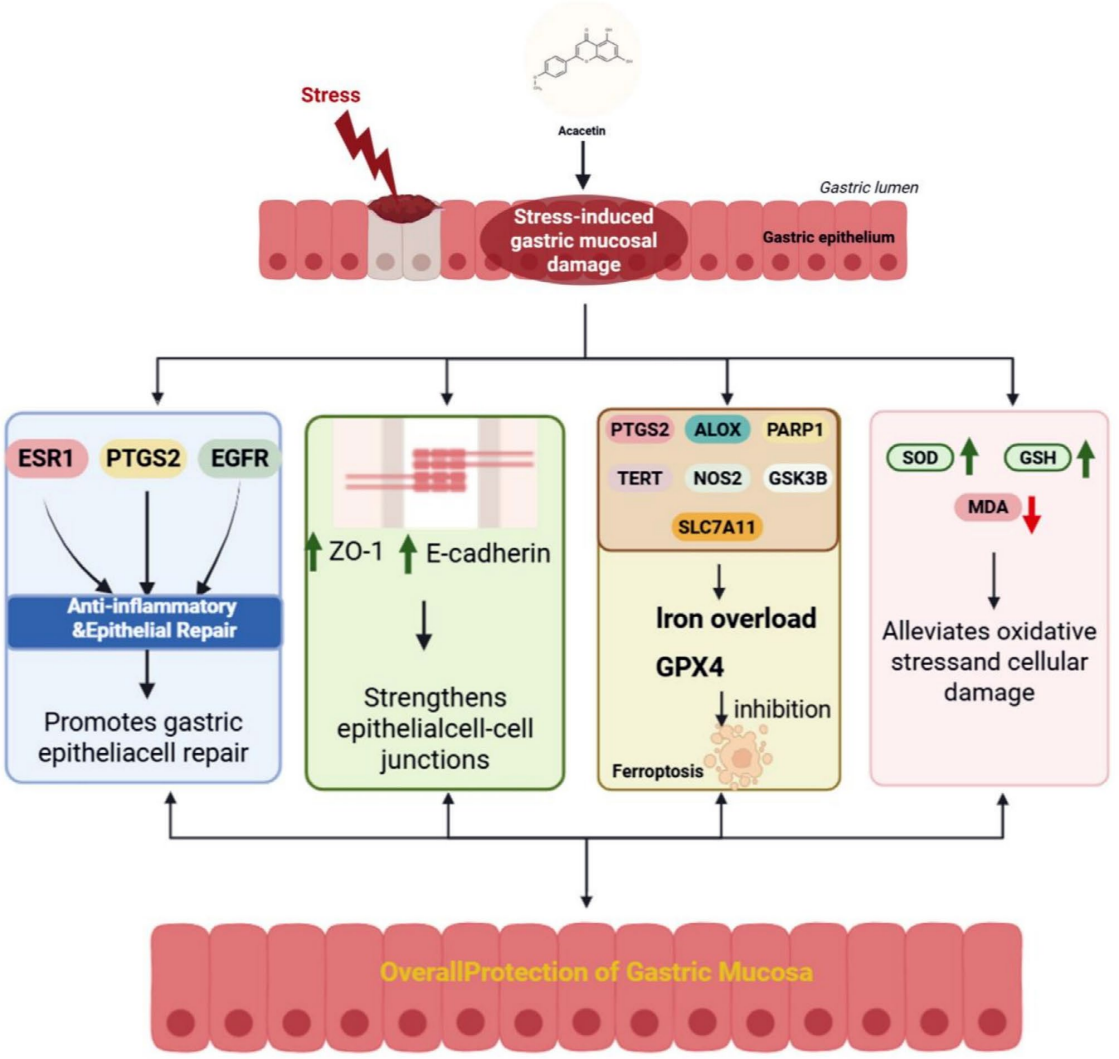
Mechanisms of acacetin against stress‐induced gastric mucosal damage. Acacetin exerts protective effects on stress‐induced gastric mucosal injury through multiple mechanisms. It attenuates inflammation and promotes epithelial repair by targeting ESR1, PTGS2, and EGFR, thereby reducing pro‐inflammatory responses and facilitating gastric epithelial regeneration. Acacetin also maintains gastric mucosal barrier integrity by upregulating tight junction protein ZO‐1 and adherens junction protein E‐cadherin, enhancing epithelial cell–cell adhesion. In addition, acacetin inhibits ferroptosis by regulating PTGS2, GSK3B, ALOX, PARP1, TERT, NOS2, and SLC7A11, leading to reduced iron overload and increased GPX4 expression, thereby suppressing lipid peroxidation–induced cell death. Furthermore, acacetin alleviates oxidative stress by decreasing MDA levels and increasing antioxidant enzyme activities, including SOD and GSH. This figure was created by https://BioRender.com. EGFR, epidermal growth factor receptor; ESR1, estrogen receptor 1; GPX4, glutathione peroxidase 4; GSH, glutathione; MDA, malondialdehyde; PTGS2, prostaglandin‐endoperoxide synthase 2; SOD, superoxide dismutase; ZO‐1, zonula occludens‐1.

In the present study, through network pharmacology, potential drug targets of acacetin were explored, and experimental validation confirmed that acacetin reduces oxidative stress‐triggered ferroptosis and gastric ulcer formation in CWIR mice. Also, 25 mg/kg/day might be the optimal therapeutic dosage of acacetin as an alternative treatment option for SGU. In summary, acacetin exhibits significant protective effects against stress‐induced gastric mucosal injury (Figure 6).

However, this study also has some limitations. First, several pharmacokinetic challenges restrict its clinical application. Specifically, acacetin exhibits notably low water solubility and poor oral absorption, and it is unstable in physiological pH conditions and gastrointestinal fluids, all of which collectively result in its extremely low oral bioavailability. Also, the studies on intravenous administration have revealed rapid plasma clearance and a brief half‐life, suggesting that sustaining therapeutic plasma concentrations could be difficult. These pharmacokinetic constraints underscore the necessity for additional research to devise optimized formulations or delivery methods aimed at enhancing the bioavailability and therapeutic potential of acacetin in clinical practice. Secondly, although network pharmacology analysis predicted the involvement of potential signaling pathways such as PI3K/AKT, EGFR, and VEGF, it did not offer direct in vivo evidence for these pathways. Thirdly, molecular docking results are based on computational predictions and have not yet been validated experimentally. Thus, further studies will experimentally verify these predicted pathways, clarify molecular mechanisms, and use techniques like SPR or CETSA for further validation of predicted interactions.

## Author Contributions

L.W. and Z.Z. conceived the idea and designed the study. L.W. wrote and revised the manuscript. X.Z., L.C., and F.Z. contributed to animal experiments and molecular biological experiments. S.C. and L.Z. performed the RNA‐seq analysis. L.C., Q.‐X.Z., and L.W. completed the molecular docking and bioinformatics analysis. All authors have read and agreed to the published version of the manuscript.

## Funding

This work was supported by the Key Project of the Science and Technology Department in Sichuan Province (No. 23ZDYF1182) for Ling Zhu, the Research Project on Cultivating Top‐notch Students in Basic Disciplines at Sichuan University (No. SCUBJ110) for Li‐hong Wan, the Key Research and Development Program of Tibet Autonomous Region Science and Technology Plan Project (No. XZ202301ZY0048G) for Shu‐ting Cheng, and the Fundamental Research Funds for the Central Universities (No. SCU2024D022).

## Consent

The authors have nothing to report.

## Conflicts of Interest

The authors declare no conflicts of interest.

## Data Availability

The data that support the findings of this study are available from the corresponding author upon reasonable request.
